# Recognition and Management of Cognitive Impairment in Chronic Obstructive Pulmonary Disease (COPD): Implications of Clinical Confidence

**DOI:** 10.3390/medicina62030438

**Published:** 2026-02-26

**Authors:** Rayan A. Siraj

**Affiliations:** Department of Respiratory Therapy, College of Applied Medical Sciences, King Faisal University, Al-Ahsa 31982, Saudi Arabia; rsiraj@kfu.edu.sa

**Keywords:** clinical confidence, cognitive impairment, chronic obstructive pulmonary disease, clinical decision-making, multimorbidity

## Abstract

Cognitive impairment is a serious comorbidity in chronic obstructive pulmonary disease (COPD), consistently associated with adverse clinical outcomes, including impaired self-management, poor treatment adherence, reduced participation in pulmonary rehabilitation, and increased risk of mortality. Despite this, it remains inconsistently recognised and insufficiently addressed during routine COPD assessment. This narrative review synthesises current evidence on the recognition and management of cognitive impairment in COPD, with a particular focus on understanding why it continues to be under-recognised and inadequately managed in clinical practice. Across care settings, cognitive concerns are commonly identified informally, assessed selectively, or deferred altogether, even when clinicians acknowledge their relevance to respiratory assessment, treatment implementation, and patient engagement. This persistent evidence–practice gap suggests the influence of factors extending beyond disease- or patient-related explanations alone. Emerging evidence indicates that clinician-level determinants, particularly clinical confidence, play a central role in shaping cognitive care practices. Limited clinical confidence appears to mediate the translation of existing knowledge and competence into clinical action, influencing decisions to initiate assessment, communicate cognitive concerns, assume clinical ownership, and pursue follow-up or referral. These confidence-related barriers are further reinforced by educational limitations, time constraints, diagnostic ambiguity, particularly in the early cognitive impairment stage, and the absence of clear operational guidance within COPD-specific frameworks. Conceptualising cognitive care through the lens of clinical confidence provides a coherent explanation for the underrecognition of cognitive impairment in COPD. It also helps account for observed variability in clinical decision-making, highlighting clinical confidence as a modifiable intermediary between knowledge, competence, and practice and a potential target for strengthening integrated, patient-centred COPD care.

## 1. Introduction

Chronic obstructive pulmonary disease (COPD) has traditionally been defined by persistent respiratory symptoms and spirometrically confirmed airflow limitation. However, contemporary evidence supports a broader conceptualisation of COPD as a heterogeneous lung condition characterised by chronic respiratory symptoms resulting from persistent abnormalities of the airways, alveoli, and/or pulmonary vasculature, confirmed by airflow limitation and/or structural or physiological pulmonary dysfunction [1,2]. This updated framework recognises that COPD is no longer a single uniform disease but rather a spectrum of pathophysiological processes with variable biological mechanisms and clinical trajectories, some of which may precede overt airflow limitation.

Within this framework, COPD is increasingly understood as a multisystem condition frequently accompanied by extrapulmonary comorbidities, including cardiovascular disease, metabolic disorders, psychological conditions, osteoporosis, and cognitive impairment [3,4]. Most individuals with COPD present with at least one comorbidity [5,6], which substantially influences clinical management [7], symptom burden, healthcare utilisation, and mortality, with non-pulmonary causes accounting for a significant proportion of deaths [8]. This multisystem perspective reflects growing recognition that systemic inflammation, vascular dysfunction, and impaired tissue repair processes extend beyond the lungs, reinforcing the need to address comorbid conditions within routine care. In this broader disease context, cognitive impairment emerges as a clinically important yet often under-recognised component of COPD management [9,10].

Cognitive impairment in individuals with COPD has been associated with adverse effects across multiple domains of disease management [9]. Cognitive deficits have been linked to increased healthcare utilisation [11], inadequate medication adherence [12], increased functional dependence [13], improper inhaler use [14], and reduced completion rates of pulmonary rehabilitation programmes [15]. Collectively, these consequences may contribute to poorer disease control, higher hospitalisation rates [12], and, in some cases, an increased risk of mortality. Accordingly, timely recognition of cognitive impairment in COPD is clinically important, as early identification may enable targeted interventions to optimise care and improve outcomes.

Estimates of cognitive impairment prevalence in COPD vary widely, ranging from approximately 4% to 61% [16,17], with evidence suggesting that up to one in four patients may be affected [18]. This variability is largely attributable to differences in study populations, methodologies, and cognitive assessment tools. Population-based data further indicate that individuals with COPD are at increased risk of developing cognitive impairment and subsequent dementia compared with those without COPD [19]. Although regional data remain limited, the rising global prevalence of COPD is likely to be accompanied by a parallel increase in the burden of associated cognitive comorbidity.

Over the past two decades, research has increasingly examined cognitive impairment in COPD, focusing on prevalence, risk factors, and underlying mechanisms. Proposed mechanisms include chronic hypoxaemia, systemic inflammation, oxidative stress, vascular dysfunction, and shared risk factors such as smoking and cardiovascular disease [9,20]. Neuroimaging studies have also demonstrated structural and functional brain changes in patients with COPD, supporting the biological plausibility of cognitive involvement [21]. Together, this evidence establishes cognitive impairment as a meaningful comorbidity with important clinical implications.

Despite this expanding evidence base, cognitive impairment remains inconsistently recognised and infrequently addressed in routine COPD practice [22], although comorbidities (including cognitive impairment) are recommended to be managed alongside COPD [23]. Disease- and patient-related explanations alone do not fully account for this gap. While cognitive impairment may present subtly and overlap with conditions such as depression and anxiety, these factors do not explain why clinicians who are aware of its relevance often fail to implement routine assessment or structured management strategies [22]. Emerging evidence suggests that clinicians commonly acknowledge the importance of cognitive impairment in COPD, yet report low confidence in recognising, assessing, and managing cognitive concerns, alongside practical barriers such as limited training, time constraints, and unclear clinical pathways.

Clinical confidence may therefore represent an important clinician-level determinant influencing whether cognitive impairment is actively addressed during COPD visits [24]. Clinical confidence encompasses clinicians’ perceived ability to recognise cognitive impairment, interpret its significance, communicate concerns with patients and families, and initiate appropriate management or referral. A lack of confidence, even in the presence of knowledge and awareness, may result in selective assessment, reliance on informal judgement, or avoidance of cognitive issues altogether. Understanding the role of clinical confidence may therefore provide insight into the persistent evidence–practice gap observed in cognitive care for patients with COPD.

This narrative review examines the role of clinical confidence in the recognition and management of cognitive impairment in COPD. Building on existing evidence, it synthesises current practice patterns, explores factors undermining clinician confidence, and discusses implications for clinical practice, education, and future research.

## 2. Methods

### 2.1. Review Design

This review was a structured narrative review aimed at exploring and clarifying the mechanisms underlying the evidence–practice gap in the recognition and management of cognitive impairment in COPD. The objective was to provide explanatory synthesis and conceptual integration across heterogeneous sources of evidence, rather than to quantify effect sizes. A narrative approach was therefore selected to allow flexible integration of empirical COPD-specific findings, clinician-focused research, guideline documents, and relevant theoretical literature within a coherent interpretative framework.

### 2.2. Literature Search Strategy

A structured literature search was conducted in PubMed/MEDLINE, Scopus, and Web of Science to identify relevant peer-reviewed publications. The search covered the period from January 2000 to December 2025 and was limited to English-language articles.

Search terms were developed iteratively and combined keywords related to:

1. COPD (“chronic obstructive pulmonary disease”, “COPD”);
2. cognitive impairment (“cognitive impairment”, “mild cognitive impairment”, “cognition”, “dementia”); and
3. clinical practice and clinician-related determinants (“recognition”, “screening”, “assessment”, “management”, “clinical confidence”, “self-efficacy”, “clinical decision-making”, “uncertainty”, “training”, “guidelines”).

To enhance coverage, the reference lists of key primary studies, reviews, and guideline documents were manually screened.

### 2.3. Eligibility Criteria and Scope

Publications were included if they met one or more of the following criteria:

1. Examined cognitive impairment in COPD populations with relevance to clinical recognition, assessment, management, or patient outcomes;
2. Investigated clinician practices, preparedness, confidence, or perceived barriers related to cognitive care;
3. Included guideline statements or consensus documents relevant to multimorbidity or cognitive management in COPD; or
4. Explored clinician-level determinants of cognitive recognition and management in related clinical contexts, particularly primary care and dementia care.

The inclusion of literature from primary care and dementia settings was undertaken intentionally. Empirical research directly examining clinical confidence within COPD care remains limited. Evidence from related contexts was therefore used to inform transferable mechanisms of diagnostic uncertainty, role ambiguity, and confidence-related decision-making, while maintaining a clear distinction from COPD-specific findings.

Publications were excluded if they lacked clinical relevance to recognition or management processes or focused exclusively on laboratory-based outcomes without implications for clinical practice.

### 2.4. Data Extraction and Narrative Synthesis

Key information was extracted regarding study design, population characteristics, clinical setting, cognitive assessment approaches, and identified clinician-level and system-level determinants of practice. Evidence was synthesised using thematic narrative integration.

Findings were organised into four domains:

1. Clinical relevance and presentation of cognitive impairment in COPD;
2. current approaches to recognition and management;
3. clinician-level determinants, including confidence and uncertainty; and
4. organisational and system-level influences on clinical decision-making.

A formal risk-of-bias assessment was not undertaken because the objective was conceptual synthesis rather than effect estimation. The framework was developed through integration of COPD-specific evidence with established theory on clinical confidence and decision-making, with the aim of generating a clinically grounded and testable model.

## 3. Cognitive Impairment in COPD: Clinical Relevance and Practice Context

### 3.1. Clinical Manifestations of Cognitive Impairment in COPD Care

Cognitive impairment refers to a decline in one or more cognitive domains, including memory, attention, and executive functioning [25]. Mild cognitive impairment (MCI) represents an early stage of cognitive decline and is commonly conceptualised as a transitional state between normal cognitive ageing and dementia, although progression to dementia is not inevitable [26]. While MCI may not initially result in overt functional dependency, it may nevertheless have clinically meaningful consequences in the context of complex chronic diseases such as COPD, where effective management depends on patients’ cognitive capacity.

In patients with COPD, cognitive impairment is not present as a single or uniform clinical condition. Instead, impairments may involve multiple cognitive domains, most commonly attention, memory, executive function, learning, and processing speed [25]. The severity and pattern of impairment vary considerably between individuals [27], contributing to the vast heterogeneity reported across studies. In routine clinical practice, cognitive impairment is often subtle, may fluctuate over time, and is not always readily apparent during brief clinical encounters [28].

Patients with COPD may appear cognitively intact during short interactions yet experience difficulties when required to perform more complex tasks, such as following multi-step instructions, adapting to changes in treatment plans, or applying health information in real-life situations. As a result, cognitive impairment is frequently inferred indirectly through patient behaviour rather than identified through a structured cognitive assessment. Clinicians may become aware of potential cognitive difficulties only after repeated misunderstandings, inconsistent symptom reporting, or apparent difficulties adhering to treatment recommendations. Consequently, cognitive impairment is often recognised later in the care pathway, when its impact on disease management has already become evident [29]. These features—subtle presentation, variability across cognitive domains, and delayed behavioural signals—contribute to the under-recognition of cognitive impairment in COPD and may undermine clinicians’ confidence in its identification and management.

### 3.2. Functional and Clinical Consequences of Cognitive Impairment for COPD Management

Cognitive impairment has important clinical implications for the management of COPD, as effective disease control depends heavily on patients’ cognitive capacity. Cognitive deficits may impair patients’ ability to recognise symptom deterioration, respond appropriately to exacerbations, and implement recommended lifestyle modifications, thereby reducing their capacity to engage independently in care and increasing reliance on caregivers and healthcare services. Even mild cognitive impairment may have clinically meaningful effects in COPD, as core components of respiratory management—including understanding treatment goals, following monitoring plans, coordinating care across multiple healthcare providers, and responding to changes in symptoms—require intact executive function and memory [20]. When these cognitive capacities are compromised, treatment effectiveness may be reduced despite appropriate prescription, education, and clinical follow-up.

These functional limitations translate into challenges across several key components of COPD care, including self-management, adherence to treatment, inhaler technique [30], and participation in pulmonary rehabilitation. Cognitive deficits have been associated with increased functional dependence [14], poorer adherence to prescribed medications [31], difficulties with smoking cessation, and reduced completion rates of pulmonary rehabilitation programmes [9]. Impairments in executive function, learning, memory, and visual processing may compromise patients’ capacity to engage effectively in self-management, a cornerstone of contemporary COPD management.

Self-management requires complex cognitive processes, including symptom monitoring, adherence to pharmacological therapy, maintenance of physical activity and healthy lifestyle behaviours, and coping with the demands of daily living. Effective self-management has been shown to improve dyspnoea and health-related quality of life and to reduce hospitalisations; however, these benefits depend on adequate cognitive functioning. In the presence of cognitive impairment, the ability to perform self-management tasks may be compromised, increasing the risk of suboptimal disease control and adverse outcomes. Although research specifically examining the impact of cognitive impairment on self-management in COPD remains limited, available evidence suggests a clinically meaningful association. A systematic review of 13 studies reported that cognitively impaired patients with COPD require greater assistance with activities of daily living, are less likely to adhere to treatment recommendations, and experience greater difficulty using inhaler devices correctly [14]. Proper inhaler technique is essential for optimising drug delivery and achieving effective disease control [32], yet cognitive impairment has been consistently linked to inhaler misuse. Lower scores on global cognitive screening tools have been shown to predict poor inhaler technique, and impairments in executive function and praxis—particularly planning and sequencing—have also been associated with inhaler errors [33].

The complexity of inhaler devices further compounds this issue, as some devices require higher levels of coordination and cognitive processing than others, potentially disadvantaging patients with cognitive impairment. Despite this, current clinical guidelines provide limited guidance on inhaler selection or optimisation for patients with COPD and coexisting cognitive impairment, leaving clinicians to rely mainly on individual judgement.

Cognitive impairment also affects engagement in pulmonary rehabilitation [11], a cornerstone of non-pharmacological COPD management that aims to improve exercise capacity, symptoms, quality of life, and clinical outcomes while reducing hospitalisation and mortality [34]. Pulmonary rehabilitation programmes incorporate exercise training, education, behavioural change, and nutritional support, all of which depend on intact cognitive abilities. Evidence indicates that patients with cognitive impairment are less likely to complete pulmonary rehabilitation programmes than cognitively intact patients. Higher dropout rates among cognitively impaired individuals suggest that deficits in cognitive functions such as planning, information processing, and impulse control may interfere with adherence and sustained engagement, thereby limiting the effectiveness of rehabilitation.

### 3.3. Challenges in Recognising Cognitive Impairment in Routine Respiratory Assessment

Several factors contribute to the underrecognition of cognitive impairment in routine COPD assessment. Cognitive symptoms often overlap with common features of COPD, including fatigue, dyspnoea, anxiety, and depression, leading to misattribution or diagnostic uncertainty [35]. Cognitive impairment may also be persistent yet subtle, making it less salient to clinicians when respiratory symptoms dominate the clinical encounter. The potential underdiagnosis of COPD itself is another issue in cognitively impaired patients. Cognitive and functional impairment may negatively affect the quality of spirometry required to confirm a diagnosis of COPD, complicating case identification and contributing to underrecognition of both conditions [36]. This bidirectional diagnostic challenge may partly explain the wide discrepancies reported in the prevalence of cognitive impairment in COPD and the heterogeneity in affected cognitive domains across studies. Differences in disease severity, degree of hypoxaemia, and the neuropsychological tests used further contribute to this variability [35].

### 3.4. Guideline Recommendations, Assessment Approaches, and Clinical Ambiguity

International COPD guidelines emphasise the importance of identifying and managing comorbidities as part of comprehensive disease management [7,37]. Cognitive impairment is recognised as a relevant comorbidity; however, guidance regarding its assessment and management remains limited. In many cases, comorbidities are recommended to be managed according to usual care pathways, without providing COPD-specific recommendations for cognitive screening, monitoring, or follow-up. This approach may inadvertently contribute to uncertainty in clinical practice.

Guidelines and evidence frameworks addressing COPD management in the context of cognitive change are particularly sparse within international literature. There is no mention of cognitive screening or structured management approaches within the Global Initiative for Chronic Obstructive Lung Disease (GOLD). A reference to cognitive screening was introduced in the 2013 American Thoracic Society/European Respiratory Society statement; however, this was limited to a review of available assessment tools rather than to recommendations for routine or systematic screening. Furthermore, the need to consider cognitive change was not addressed in the clinical practice guideline update on the diagnosis and management of stable COPD developed by the American College of Physicians, American College of Chest Physicians, American Thoracic Society, and European Respiratory Society. Collectively, these omissions leave cognitive assessment poorly defined across major guideline frameworks.

From a clinical perspective, appropriate intervention strategies for cognitive impairment in COPD begin with timely recognition. Importantly, assessing cognitive function is not complex and does not require advanced equipment. Several validated screening tools are available and can be administered in routine clinical practice, typically within 5–15 min, providing an overview of whether patients exhibit signs of cognitive impairment warranting referral for comprehensive assessment [38]. Despite this, cognitive screening is not routinely incorporated into COPD visits.

The wide availability of screening tools may pose a challenge in itself. Clinicians may be uncertain about which tools are most appropriate for patients with COPD, whether brief global screening is sufficient, or whether more comprehensive domain-specific assessment is required. Moreover, it remains unclear whether cognitive screening should be applied universally to all patients with COPD or selectively to those presenting with overt cognitive or functional difficulties. This lack of clarity may contribute to inconsistent practice and reliance on informal clinical judgement.

To enhance practical applicability within routine COPD care, a structured yet flexible screening approach is warranted (Table 1). In time-constrained respiratory consultations, brief validated tools such as the Mini-Cog or the General Practitioner Assessment of Cognition (GPCOG) may serve as initial screening instruments due to their short administration time and minimal training requirements. More comprehensive tools, including the Montreal Cognitive Assessment (MoCA), may be reserved for patients in whom initial screening raises concern or for those with subtle cognitive deficits. Rather than universal screening, a selective trigger-based strategy—guided by observable indicators such as repeated inhaler misuse, inconsistent clinical history, unexplained nonadherence, frequent exacerbation-related healthcare utilisation, or early disengagement from pulmonary rehabilitation—may enhance feasibility while preserving clinical efficiency. Embedding cognitive prompts within annual COPD reviews, inhaler education sessions, or pulmonary rehabilitation intake assessments may further standardise consideration without substantially increasing consultation burden.

Consequently, cognitive impairment—particularly in its early stages, such as mild cognitive impairment—may remain underdiagnosed, with potentially significant consequences for clinical outcomes. The lack of explicit guidance regarding screening responsibility, assessment frequency, and referral pathways perpetuates uncertainty around clinical expectations and contributes to inconsistent practices across care settings.

### 3.5. COPD-Specific Evidence on Recognition and Practice Gaps

Although empirical investigations directly measuring clinical confidence in COPD populations remain limited, condition-specific research consistently demonstrates variability in the recognition and management of cognitive impairment within respiratory practice. Survey-based data from respiratory physicians indicate that while most clinicians acknowledge the clinical impacts of cognitive impairment in COPD, routine cognitive screening is not systematically implemented during standard respiratory assessments [24]. Cognitive evaluation is instead selective, typically prompted by overt functional decline rather than embedded within structured care pathways. This discrepancy between awareness and routine assessment highlights a measurable implementation gap within pulmonary services themselves, rather than an issue inferred from other specialities.

Additional studies further illustrate how cognitive impairment directly influences respiratory management processes, yet remains under-integrated into clinical workflows. Impairments in executive function, memory, and attention have been shown to compromise the acquisition and retention of inhaler technique, thereby affecting medication delivery and disease control [12,14,32]. Similarly, cognitive deficits have been associated with reduced adherence to pharmacological regimens and higher dropout rates from pulmonary rehabilitation programmes [15,39]. Despite these established associations, structured cognitive assessment is rarely embedded into pulmonary rehabilitation intake protocols or inhaler education sessions. This disconnect between documented clinical impact and routine assessment practices suggests that barriers exist at the level of implementation rather than knowledge alone. The persistence of such patterns within COPD populations strengthens the need for a structured explanatory framework specific to respiratory care.

Structural features of current respiratory practice may further contribute to variability in cognitive recognition. Guideline frameworks acknowledge the importance of multimorbidity management but offer limited procedural guidance on operationalising cognitive concerns in routine COPD consultations. While the conceptual relevance of comorbidities is widely accepted, specific clinical prompts, documentation expectations, and follow-up pathways related to cognitive change remain insufficiently defined. In the absence of structured workflow integration, clinicians are left to rely on individual judgement when determining whether a cognitive assessment is warranted. This lack of operational clarity may unintentionally position cognitive evaluation as secondary to immediate respiratory priorities. Consequently, cognitive impairment may be acknowledged in principle while remaining variably addressed in everyday clinical encounters. These structural ambiguities help explain why recognition and management patterns differ across practitioners and settings.

Taken together, the available COPD literature confirms that cognitive impairment meaningfully affects disease management yet is not systematically addressed within respiratory workflows [9,22,35,40]. While direct empirical evaluation of clinical confidence within COPD care is limited, persistent variability in screening, adaptation, and referral behaviours suggests the influence of clinician-level determinants beyond knowledge alone. Clinical confidence is therefore positioned as a structured and testable explanatory construct for this practice variability, offering a behaviourally grounded direction for empirical investigation within COPD care.

## 4. Clinical Confidence: Conceptual Foundations

### 4.1. Defining Clinical Confidence in Healthcare Practice

Confidence is commonly defined as a mental attitude of trust or assurance in a person, judgement, or course of action, reflecting a belief in one’s ability to respond appropriately to a given situation [41]. Rather than representing a fixed personality trait, confidence is increasingly understood as a dynamic and context-sensitive construct that fluctuates in response to task demands, situational uncertainty, perceived consequences, and prior experience. As such, confidence plays a central role in shaping behaviour [42], influencing how individuals interpret information, tolerate ambiguity, and decide whether to act, delay, or seek further input.

A key challenge in conceptualising confidence lies in its imperfect alignment with actual ability. Empirical evidence consistently shows that individuals’ self-assessments of skills are often inaccurate [12], leading to a mismatch between confidence and competence. This miscalibration has important implications for performance and safety, particularly in high-stakes professional environments. Underconfidence may lead to hesitation, delayed decision-making, or avoidance of responsibility even when action is clinically indicated, whereas overconfidence may foster premature decisions, reduced vigilance, or failure to recognise limitations. In both cases, the consequences extend beyond individual behaviour to influence clinical outcomes, efficiency, and risk.

Beyond its immediate impact on decision-making, confidence also shapes learning and self-regulatory processes. While self-regulation has been proposed as a mechanism for improving performance, it presupposes an accurate awareness of one’s capabilities and limitations [43]. When confidence is poorly calibrated, self-regulation becomes compromised. Underconfident individuals may devote excessive effort to confirming knowledge they already have or seeking reassurance, while overconfident individuals may overlook knowledge gaps, resist feedback, or miss opportunities for professional development. Safe and effective practice, therefore, depends not on high confidence per se, but on an appropriate level of confidence that is proportionate to training, experience, and task complexity.

Within healthcare, confidence assumes particular significance due to the inherent uncertainty, cognitive demands, and potential for harm that characterise clinical decision-making. Clinical practice frequently requires action in the absence of complete information, often under time pressure and with consequences that may affect patient safety, professional accountability, and ethical responsibility. In this context, clinical confidence can be understood as a clinician’s perceived capacity to initiate, justify, and sustain clinical actions in situations characterised by diagnostic ambiguity, competing priorities, and professional risk [44]. Importantly, clinical confidence does not imply certainty or infallibility; rather, it reflects a form of professional assurance that enables responsible action despite uncertainty.

Clinical confidence operates at the intersection of cognitive, emotional, and social processes. It is influenced by multiple interacting factors, including stress [45], emotional responses [46], tolerance for uncertainty [47], cognitive load [48], group dynamics [43], organisational culture, and the clarity of clinical guidance. These influences shape how clinicians perceive their role, assess their readiness to act, and anticipate the consequences of their decisions. As a result, confidence is often task- and domain-specific rather than global. Clinicians may demonstrate high confidence in managing well-defined, protocol-driven aspects of care, yet experience diminished confidence when addressing complex, poorly defined, or extended beyond traditional biomedical boundaries.

Viewed through this lens, clinical confidence functions as a behavioural threshold within healthcare practice—one that determines whether knowledge is translated into assessment, assessment into action, and action into sustained management. Where confidence is eroded by uncertainty, lack of guidance, or perceived risk, even robust evidence may fail to influence practice. Conceptualising confidence as a multidimensional, situational construct is therefore essential for understanding gaps between knowledge and care delivery, particularly in complex chronic conditions where clinical pathways are less explicit, and decision-making extends beyond physiological management.

### 4.2. Interrelationships Between Clinical Confidence, Competence, and Knowledge

Clinical competence is a widely used, multidimensional concept encompassing a range of attributes rather than a single capability. Holistic conceptualisations describe competence as an integration of knowledge, skills, and attitudes, and, in some definitions, extend to abilities, values, judgement, and performance [44,45] (Table 2). Within healthcare practice, competence therefore reflects the capacity of clinicians to integrate these attributes to meet accepted professional standards and fulfil clinical responsibilities.

The importance of competence in healthcare delivery is well established. Health systems analyses have demonstrated that deficiencies in care quality persist across settings, with inadequate provider competence identified as a major contributor to poor outcomes [49]. Competent care has been defined as involving systematic patient assessment, accurate diagnosis, appropriate treatment or preventive intervention, and effective patient counselling [50]. Improving provider competence is therefore regarded as a key strategy for enhancing care quality and health outcomes, reinforcing the principle that competent care begins with competent clinicians.

Knowledge represents a core component of clinical competence [51]. Inadequate knowledge has been consistently linked to poor-quality care, and multiple strategies—such as training, mentoring, and supervision [52]—have been implemented to address knowledge gaps. However, despite improvements in education and training, variability in clinical practice remains evident, suggesting that knowledge and competence alone do not fully explain how care is delivered in routine settings.

Clinical confidence plays a critical role in shaping this relationship. To be competent in practice, clinicians must not only possess knowledge and skills but also feel sufficiently confident in their ability to perform clinical tasks. Evidence across healthcare settings demonstrates that confidence is associated with provider behaviour [53,54], influencing whether clinicians initiate and carry out indicated interventions. More confident clinicians have been shown to perform recommended clinical actions more consistently [54], whereas lower confidence may lead to hesitation or the omission of care.

**Figure 1:**
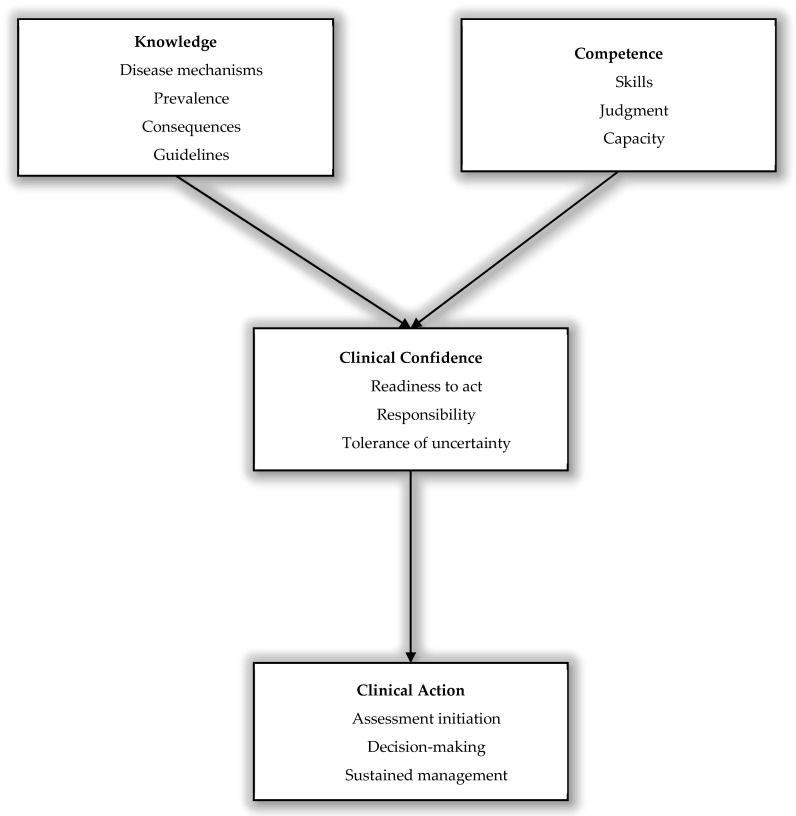
Conceptual framework illustrating clinical confidence as a determinant of cognitive assessment and management. This conceptual model illustrates how clinicians’ knowledge (e.g., disease mechanisms, consequences, and evidence) and clinical competence (e.g., skills, judgement, and performance capacity) contribute to clinical confidence, defined as perceived readiness to act, a sense of responsibility, and tolerance of uncertainty. Clinical confidence mediates the translation of knowledge and competence into clinical action, including initiation of assessment, decision-making, and sustained clinical management.

Taken together, these observations indicate that competence and knowledge, while essential, are not sufficient on their own to ensure consistent clinical performance. Clinical confidence appears to influence whether competence is translated into action, highlighting its role as a critical intermediary between capability and practice (Figure 1).

## 5. Recognition and Management of Cognitive Impairment in COPD: Current Practice

### 5.1. Informal and Selective Recognition of Cognitive Impairment in Routine COPD Assessment

In routine clinical practice, cognitive impairment in patients with COPD is most commonly recognised informally rather than through structured cognitive assessment. Clinicians frequently infer cognitive difficulties from observable behaviours during consultations, such as difficulty understanding instructions, communication problems, or repeated challenges in disease management, rather than administering validated cognitive screening tools. Formal cognitive assessment is not routinely embedded within respiratory evaluations, despite the availability of brief, feasible instruments.

Screening practices are typically selective and symptom-driven. Cognitive assessment is more likely to be considered when cognitive problems are overt or when management difficulties arise, rather than as part of a systematic approach. Evidence from clinician surveys indicates that although most clinicians acknowledge the negative impact of cognitive impairment on COPD outcomes, a substantial proportion do not perceive routine screening as necessary. This selective approach may contribute to underrecognition of early cognitive impairment, particularly mild cognitive impairment, which may not be clinically apparent but can nonetheless influence assessment accuracy and management.

This practice pattern is particularly relevant in the context of respiratory investigations. Cognitive impairment may compromise patients’ ability to perform spirometry manoeuvres correctly or to understand exercise testing instructions, potentially resulting in inaccurate estimates of lung function or exercise capacity. Despite this, cognitive function is rarely assessed alongside respiratory testing, suggesting a disconnect between recognised functional implications and clinical practice.

### 5.2. Management, Communication, and Referral Pathways in Cognitive Assessment

When cognitive impairment is suspected or identified in patients with COPD, subsequent management responses are often inconsistent. Clinicians infrequently report systematically adapting treatment strategies based on cognitive status, such as simplifying therapeutic regimens, selecting appropriate inhalers, or tailoring educational approaches. Instead, cognitive impairment is commonly treated as a contextual background factor rather than an active determinant guiding clinical decision-making.

Communication of cognitive concerns with patients and families similarly remains variable and cautious. Clinicians may hesitate to discuss cognitive impairment explicitly, particularly in the absence of a definitive diagnosis or a clearly defined management pathway. Concerns related to stigma, patient distress, and uncertainty regarding how to explain cognitive findings may result in indirect, delayed, or absent communication. Consequently, patients and caregivers may remain unaware of cognitive difficulties that directly affect understanding, adherence, and engagement with treatment. Although communication problems and misunderstandings are known contributors to nonadherence in COPD, including forgetting or refusing prescribed therapy, these challenges are not consistently framed as cognitive concerns during clinical encounters. This limits opportunities to involve caregivers, corroborate symptoms through close informants, and implement supportive strategies that could mitigate the impact of cognitive impairment on disease management.

Referral practices for cognitive impairment in COPD are likewise heterogeneous and lack standardisation. Decisions to refer for further cognitive evaluation are typically based on individual clinician judgement, perceived severity of impairment, and local service availability. Mild or early cognitive impairment is less likely to prompt referral, particularly when respiratory symptoms dominate clinical priorities or when clinicians are uncertain about the benefits of further assessment. Although cognitive screening is generally conceptualised as an initial step toward comprehensive evaluation, uncertainty regarding referral thresholds and professional responsibility may lead to delayed or omitted escalation.

The absence of clear care pathways integrating respiratory services with cognitive assessment and specialist follow-up further contributes to fragmented care. This lack of integration limits the incorporation of cognitive findings into ongoing COPD management and reinforces practice variability. Substantial differences are observed across healthcare professionals and care settings, influenced by variation in training exposure, clinical workload, consultation time, and access to multidisciplinary services. Evidence suggests that only a minority of clinicians receive specific training in managing cognitive impairment in COPD, which may reinforce reliance on informal recognition and selective assessment. Although targeted attention to higher-risk groups is often recommended, the lack of operational guidance leads to inconsistent application across practitioners and settings.

## 6. Evidence of Clinical Confidence Gaps in Cognitive Management

### 6.1. Hesitation to Initiate Cognitive Assessment as a Manifestation of Confidence Gaps

A recurring theme across studies is clinicians’ hesitation to initiate cognitive assessment, despite acknowledging the clinical relevance of cognitive impairment. Within the COPD context, recent findings show that although most clinicians recognise the negative impact of cognitive impairment on COPD outcomes, only a proportion actively seek to identify it during routine assessment, and even fewer use validated screening tools [24]. This discrepancy suggests that hesitancy reflects a gap between recognising importance and initiating assessment in practice, rather than a lack of awareness.

Current literature suggests that this hesitation is closely linked to multiple, interrelated barriers. Findings from primary care dementia research demonstrate that underdiagnosis is common, with approximately 50% of early cases missed [55]. Contributing factors include patient hesitancy driven by stigma, under-recognition or misattribution of early cognitive symptoms, substantial demands on provider time, and—critically—the lack of confidence among clinicians in diagnosing and treating cognitive impairment [55,56,57]. Together, these factors create a clinical context in which initiating cognitive assessment is perceived as complex, time-consuming, and potentially difficult to manage.

When clinicians anticipate that cognitive assessment may uncover impairment in the absence of clear management pathways or feasible follow-up options, confidence in initiating assessment is reduced. Under-recognition of symptoms further reinforces this hesitation, particularly when cognitive changes are subtle or attributed to ageing or comorbid illness. In time-pressured consultations, clinicians may therefore defer cognitive evaluation, prioritising immediate respiratory concerns and postponing assessment until impairment becomes more overt.

Importantly, this pattern of hesitation is not unique to COPD. Evidence from primary care and dementia care consistently demonstrates similar reluctance to initiate cognitive assessment, even when screening tools are available and cognitive impairment is recognised as clinically important [58,59]. Across these settings, variability in screening behaviour is less reflective of differences in evidence than of clinicians’ confidence, training exposure, and tolerance of diagnostic uncertainty. The consistency of this pattern across disease contexts suggests that hesitation represents a broader confidence-related behavioural response to uncertainty, rather than a condition-specific knowledge deficit.

Collectively, these findings suggest that hesitation to initiate cognitive assessment in COPD reflects a confidence-related behavioural response shaped by stigma, symptom ambiguity, time constraints, and uncertainty regarding diagnosis and treatment. In the absence of sufficient confidence and structural support, cognitive assessment is more likely to be delayed than proactively pursued, reinforcing under-detection and reactive care pathways.

### 6.2. Discomfort with Disclosure, Clinical Ownership, and Follow-Up Responsibilities

Clinical confidence gaps are particularly evident in clinicians’ discomfort discussing cognitive impairment with patients and families. Even when cognitive impairment is suspected or identified, clinicians frequently report uncertainty about how to initiate and navigate these conversations, especially in the absence of a definitive diagnosis or clearly established care pathways. Concerns related to stigma, patient distress, and the perceived consequences of labelling cognitive impairment may further discourage open discussion, leading to delayed or avoided disclosure.

Current literature indicates that this discomfort with cognitive conversations is not unique to COPD [58,60]. Primary care clinicians, who are typically the first point of contact for patients with cognitive concerns and often view themselves as on the frontline of brain health, consistently report hesitation to raise cognitive concerns. A large systematic review examining provider–patient brain health conversations identified widespread reluctance among clinicians to initiate discussions about cognitive change, alongside parallel hesitation from patients to disclose symptoms [61]. Importantly, the review highlighted uncertainty about how to frame concerns, limited guidance on next steps, and the influence of social and cultural factors on engagement, all of which contribute to missed or delayed conversations even before formal screening or diagnosis.

Within the COPD context, similar dynamics appear to shape clinical behaviour. Survey-based evidence indicates that many clinicians report inadequate training in recognising and managing cognitive impairment in COPD, which is closely associated with low confidence in discussing cognitive concerns with patients and families [24]. In the absence of clear communication frameworks or shared language for disclosure, clinicians may avoid raising cognitive issues altogether or rely on indirect cues rather than explicit discussion.

Taken together, these findings suggest that under-recognition of cognitive impairment in COPD is not solely a matter of detection, but also reflects clinicians’ discomfort with disclosure itself. Hesitant communication, uncertainty about how to discuss cognitive concerns, and fear of negative consequences may all undermine timely and transparent conversations, reinforcing delayed recognition and fragmented care.

### 6.3. Illustrative Clinical Scenario: Confidence Gap in Practice

Consider a 74-year-old patient with moderate COPD who repeatedly demonstrates incorrect inhaler technique despite prior education. The clinician suspects possible executive dysfunction but hesitates to initiate formal cognitive screening due to uncertainty regarding tool selection, result interpretation, referral pathways, and consultation time constraints. In the absence of structured assessment, inhaler education is repeated without adaptation, and caregiver involvement is not explored. Over subsequent visits, adherence remains inconsistent and exacerbation frequency increases. In this scenario, awareness of the potential cognitive contribution is present; however, limited confidence in initiating assessment and managing its consequences leads to deferral of action. Structured screening tools, clearly defined referral pathways, and workflow prompts could reduce uncertainty and support earlier adaptation of care (Table 3).

## 7. Factors Undermining Clinical Confidence in Cognitive Care for COPD

### 7.1. Educational and Training Deficits as Key Drivers of Low Clinical Confidence

Educational and training deficits represent a central structural factor undermining clinicians’ confidence in cognitive care for patients with COPD. While cognitive impairment is widely recognised as clinically relevant, many clinicians report limited formal education on its recognition, assessment, and management within respiratory assessment. This gap restricts the development of practical competencies and contributes to uncertainty when translating knowledge into clinical action.

Evidence across healthcare settings consistently links inadequate training to reduced clinician confidence in cognitive assessment. Findings from systematic reviews showed that limited knowledge and lack of trained providers are key, modifiable barriers to cognitive screening and management, highlighting education as a prerequisite for confident engagement [62,63,64]. Similar patterns are reported in primary care, where many physicians describe insufficient preparation to identify early cognitive impairment and uncertainty regarding management strategies [65]. International studies further demonstrate limited familiarity with early cognitive decline among general practitioners, with nearly two-thirds reporting low confidence in diagnosing and managing cognitive impairment [66,67]. These observations reinforce the view that confidence deficits in cognitive care are not unique to COPD but reflect a broader educational shortfall in integrating cognitive assessment into chronic disease management.

In the context of COPD, these educational gaps appear particularly pronounced. A large national survey of physicians in Saudi Arabia demonstrated that only a small minority reported receiving adequate training in the recognition and management of cognitive impairment in COPD, with more than 80% indicating insufficient preparation [24]. Despite high awareness of the clinical impact of cognitive impairment, inadequate training was associated with persistent uncertainty in assessing, interpreting, and discussing cognitive concerns.

Notably, clinicians consistently identify education-related factors—poor training, limited knowledge, and absence of structured guidance—not only as barriers to effective practice but also as key enablers of improvement. This alignment underscores education and training as foundational, modifiable determinants of clinical confidence and highlights their critical role in supporting consistent, proactive cognitive care in COPD.

Beyond recognising these deficits, structured integration of cognitive assessment training into respiratory curricula and continuing professional development is warranted. Educational programmes should move beyond theoretical awareness and incorporate practical training in cognitive screening, result interpretation, and management pathways within routine COPD consultations. Such targeted training may strengthen both technical competence and clinician confidence, enabling more consistent and proactive cognitive care.

### 7.2. Competing Clinical Priorities, Time Constraints, and Diagnostic Ambiguity

Time pressure and competing clinical priorities are significant constraints on clinicians’ confidence in addressing cognitive impairment [68,69]. In routine COPD assessment and management, consultations are often dominated by respiratory symptom management, exacerbations, and treatment optimisation, leaving limited time for broader cognitive assessment. Within these constrained timeframes, cognitive evaluation may be perceived as an additional task that is difficult to integrate into routine practice. This persistent pressure can discourage clinicians from initiating cognitive discussions, contributing to selective assessment and reinforcing uncertainty about when and how cognitive concerns should be addressed.

Diagnostic ambiguity further compounds these challenges [70]. Cognitive impairment in COPD is heterogeneous and often subtle, particularly in its early stages. Clinicians may be uncertain about how to interpret borderline screening results, distinguish cognitive impairment from fatigue or psychological distress, or determine the clinical significance of mild deficits [71]. Fear of misclassification and concern about labelling patients incorrectly may reduce confidence in initiating assessment, particularly in the absence of disease-specific cognitive tools or clear diagnostic thresholds.

These challenges highlight the need for brief, validated, and clinically feasible cognitive screening tools that have been specifically evaluated in COPD populations. Tools that are easy to administer during time-constrained respiratory consultations and can distinguish cognitive deficits from overlapping symptoms such as depression or fatigue may improve both diagnostic clarity and clinician confidence.

These uncertainties are amplified by guideline-level ambiguity. Although international COPD guidelines emphasise the importance of recognising comorbidities, they provide limited operational guidance on identifying, assessing, or monitoring cognitive impairment. Without clear recommendations, clinicians may perceive cognitive assessment as optional rather than integral, reinforcing hesitation and reducing confidence in decision-making.

### 7.3. Role Uncertainty, System-Level Barriers, and Organisational Constraints

Role uncertainty emerges as a foundational challenge shaping clinicians’ engagement with cognitive impairment in COPD care. Many clinicians operate within care models that prioritise physiological stability, symptom control, and exacerbation prevention, leaving cognitive assessment positioned ambiguously within clinical responsibilities. When it is unclear whether respiratory teams should lead cognitive management, clinicians may hesitate to initiate assessment or management. This lack of clearly assigned responsibility weakens professional ownership and fosters a tendency to defer cognitive concerns to other services, even in the absence of explicit referral pathways. As a result, cognitive impairment may remain acknowledged in principle but unaddressed in practice.

System-level barriers further compound this uncertainty by limiting the structural capacity required to support cognitive management. In the absence of standardised protocols or embedded assessment pathways, clinicians are left to rely on individual judgement rather than institutional guidance. This variability creates inconsistency in practice and undermines confidence, particularly when clinicians are unsure how to proceed after identifying cognitive concerns. Without clear processes for documentation, referral, follow-up, and interdisciplinary communication, cognitive impairment becomes difficult to integrate into routine COPD assessment.

Organisational constraints related to workload and resource allocation intensify these challenges. High patient volumes, limited staffing, and competing clinical priorities constrain the time available for comprehensive assessment. Cognitive impairment, which often requires careful observation, longitudinal evaluation, and nuanced communication, may be perceived as incompatible with the pace and structure of routine respiratory clinics. Even clinicians who recognise cognitive concerns may feel unable to pursue them adequately, reinforcing a pattern in which cognitive management is deferred or omitted due to practical limitations rather than lack of awareness.

The physical and organisational environment in which care is delivered also plays a critical role. Effective discussion of cognitive changes often requires privacy, continuity, and a supportive setting that facilitates engagement with both patients and caregivers. When clinical environments do not allow for confidential or unhurried conversations, clinicians may be reluctant to raise sensitive topics related to cognition, memory, or functional decline with patients or family members. These environmental limitations not only restrict communication but may also contribute to discomfort or uncertainty when addressing issues perceived as personal or potentially distressing.

Patient-related factors interact with these systemic and organisational constraints, further eroding clinician confidence. Many patients prioritise the management of respiratory symptoms, perceiving cognitive changes as less urgent or unrelated to their pulmonary condition. This framing influences clinical encounters, particularly when time is limited, and visit agendas are driven by symptom burden. In such contexts, clinicians may be uncertain about introducing cognitive concerns that patients have not explicitly raised, especially when cognitive impairment is not yet severe or is perceived as outside the patient’s expectations of respiratory assessment.

Taken together, role ambiguity, system-level deficiencies, organisational pressures, and patient-driven dynamics create a clinical environment that discourages proactive engagement with cognitive impairment (Table 4). Clinicians may recognise the relevance of cognitive impairment to COPD outcomes, yet lack the clarity, support, and practical conditions required to address it confidently. This disconnect between awareness and action reinforces the marginalisation of cognitive care within COPD management and highlights the need for clearer role delineation, supportive organisational structures, and integrated care pathways to enable confident and consistent clinical practice.

## 8. Conceptual Contribution and Future Directions

This review advances the literature by reframing the under-recognition of cognitive impairment in COPD through an implementation-oriented perspective focused on clinician decision-making within respiratory practice. A structured conceptualisation of clinical confidence as a behavioural mediator of cognitive care implementation within COPD practice has not previously been articulated in the literature. Although prior studies have described prevalence, mechanisms, and clinical consequences, fewer have articulated a structured behavioural explanation for how cognitive impairment is operationalised within routine respiratory care. By integrating condition-specific practice variability with established theory on confidence, uncertainty tolerance, and behavioural thresholds in clinical action, this manuscript develops an explanatory framework grounded in respiratory medicine. Rather than revisiting biological mechanisms, the focus shifts to understanding why cognitive impairment remains inconsistently operationalised despite clear clinical relevance. Therefore, clinical confidence is positioned as a potentially modifiable intermediary between competence and observable practice behaviours within COPD settings.

The proposed framework is intended to guide empirical investigation within respiratory populations. Clinical confidence in COPD care may be operationalised as task-specific perceived readiness to initiate screening, interpret cognitive findings, communicate concerns to patients and caregivers, and assume follow-up responsibility in the face of diagnostic uncertainty. Conceptualising confidence in relation to discrete clinical tasks allows alignment between measurable perceptions and observable behaviours. Adaptation of validated self-efficacy and confidence-alignment instruments to respiratory contexts would permit quantification of clinician confidence across these domains. Linking such measures to documented screening frequency, inhaler education modification, pulmonary rehabilitation engagement strategies, and referral initiation would allow empirical testing of the framework within COPD practice. Vignette-based simulation studies may further clarify how clinicians respond to cognitive ambiguity during respiratory consultations.

Beyond cross-sectional assessment, longitudinal and interventional research designs are required to evaluate causal pathways. Educational interventions integrated into respiratory training programmes could examine whether improvements in task-specific confidence are associated with sustained changes in cognitive assessment behaviour. Implementation studies embedding structured cognitive prompts within respiratory workflows may assess whether organisational supports modify both clinician confidence and observable practice patterns. Mixed-methods research incorporating qualitative inquiry would further illuminate how clinicians interpret responsibility, uncertainty, and role boundaries in cognitive care. Measuring confidence alongside behavioural outcomes over time would clarify whether it functions as a mediator between training exposure, structural supports, and clinical practice. These approaches collectively move the framework from conceptual integration toward empirically testable implementation research within COPD populations.

The scientific contribution of this review, therefore, lies in bridging documented COPD-specific practice variability with a structured behavioural explanation and an actionable research agenda. The framework does not presume that confidence is the sole determinant of inconsistency but identifies it as a plausible and modifiable contributor, warranting systematic evaluation. Establishing empirical support in respiratory settings would inform targeted educational, organisational, and guideline-level strategies aimed at integrating cognitive assessment into routine COPD management. By explicitly linking condition-specific evidence, behavioural theory, and methodological direction, the manuscript extends the field beyond descriptive recognition toward structured implementation inquiry.

## 9. Implications for Clinical Practice, Education, and Research

### 9.1. Integrating Cognitive Considerations into Routine COPD Care

The evidence reviewed supports positioning cognitive impairment as an active determinant of COPD management rather than a background comorbidity. In respiratory practice, this means recognising that cognitive status can directly influence the reliability of assessment, the acquisition of inhaler technique, the implementation of action plans, and engagement in pulmonary rehabilitation. Rather than relying solely on symptom severity to guide care, clinicians may consider cognitive functioning when interpreting inconsistencies in adherence or disease control. Practical integration does not require universal screening, but it does require structured awareness during consultations. A trigger-based approach, such as considering cognitive assessment when repeated inhaler misuse, inconsistent history reporting, or early rehabilitation dropout occurs, may enhance feasibility without imposing excessive burden. By linking cognitive consideration to observable management challenges, assessment becomes clinically purposeful rather than theoretical.

When cognitive concerns are suspected, management adaptation should accompany recognition. This may include simplifying treatment regimens when clinically appropriate, reinforcing education using teach-back methods, selecting inhaler devices with lower coordination demands, and involving caregivers in care planning. Documenting cognitive findings within respiratory records can facilitate continuity and interdisciplinary communication. Clarifying referral pathways to primary care, geriatrics, neurology, or memory services according to local structures reduces role ambiguity and supports coordinated follow-up. Importantly, respiratory clinicians retain responsibility for adapting COPD management in response to cognitive findings even when specialist referral is pursued. Embedding these practices within routine workflows strengthens the practical relevance of cognitive assessment in respiratory settings.

### 9.2. Educational and System-Level Strategies to Support Consistent Practice

Improving consistency in cognitive care requires targeted educational reinforcement within respiratory training programmes. Training should extend beyond familiarity with screening tools to include interpretation of results, communication strategies for discussing cognitive change, and decision-making regarding referral thresholds. Simulation-based learning and case-based discussions may help clinicians develop comfort navigating diagnostic ambiguity in COPD consultations. Continuing professional development initiatives can further reinforce cognitive-aware care among practising clinicians. Aligning respiratory curricula with multimorbidity management principles may also reduce the artificial separation between pulmonary and cognitive domains. Strengthening these competencies directly supports the confidence-behaviour pathway proposed in this review.

At the system level, organisational supports are essential to sustain change. Integrating structured prompts into electronic health records, establishing clear documentation standards, and defining shared referral responsibilities can reduce uncertainty in routine practice. Clinical pathways that clarify escalation steps following positive cognitive screens may enhance both confidence and follow-through. Allowing adequate consultation time for complex multimorbidity discussions further supports meaningful patient engagement. Future research should therefore evaluate not only educational interventions but also structural modifications within respiratory services. Investigating how workflow integration influences screening uptake, communication quality, and patient outcomes will be critical to advancing implementation efforts.

Interdisciplinary collaboration represents an important structural mechanism for strengthening cognitive care within COPD management. Clear delineation of roles between respiratory physicians, primary care providers, nurses, geriatricians, psychologists, and pulmonary rehabilitation teams may reduce ambiguity regarding screening responsibility and follow-up ownership. Pulmonary rehabilitation programmes, in particular, provide a structured platform for incorporating cognitive screening, caregiver engagement, and tailored educational adaptation. Shared-care pathways and defined escalation processes may enhance clinician confidence by clarifying when and how cognitive concerns should be addressed across services. Embedding cognitive assessment within coordinated team-based models shifts responsibility from individual discretion to structured system design, thereby promoting consistency and sustainability in practice.

Structured collaborative models used in other chronic conditions may provide a useful template. For example, integrated care pathways in cognitive impairment management incorporate routine screening, predefined referral thresholds, and shared responsibility across multidisciplinary teams. These models reduce individual clinician uncertainty by embedding cognitive assessment within established workflows rather than leaving it to discretionary judgement. Adapting similar structured pathways within respiratory services may enhance clarity, distribute responsibility, and strengthen clinician confidence in addressing cognitive concerns as part of comprehensive COPD care.

Overall, narrowing the gap between evidence and routine cognitive care in COPD requires alignment across clinician behaviour, training structures, and organisational design. Addressing confidence as a modifiable intermediary provides a pragmatic mechanism through which education and system-level strategies may translate into consistent practice. By combining applied clinical adjustments with structured research inquiry, the field can move beyond descriptive recognition toward measurable implementation progress.

## 10. Conclusions

This narrative review highlights cognitive impairment as a clinically important yet inconsistently addressed component of COPD management. Although clinicians broadly recognise its relevance to assessment accuracy, treatment adherence, and patient engagement, its integration into routine respiratory practice remains variable. Cognitive care is often characterised by informal recognition, selective assessment, hesitant communication, and fragmented referral pathways, reflecting a persistent gap between evidence and everyday clinical practice.

A key insight of this review is that this gap is driven less by lack of awareness than by limitations in clinical confidence. Insufficient training, diagnostic ambiguity—particularly in early cognitive impairment—competing clinical priorities, and unclear roles and care pathways collectively undermine clinicians’ confidence in assessing, communicating, and managing cognitive concerns. Viewed through this lens, clinical confidence functions as a key mediator between knowledge and action in COPD cognitive care. Strengthening confidence, therefore, requires system-level strategies that legitimise cognitive care, clarify responsibilities, and support practical implementation alongside education, offering a pragmatic pathway toward more integrated and patient-centred COPD management.

## Figures and Tables

**Table 1:** Practical Cognitive Screening Tools for Routine COPD Care.

Tool	Time	Strength	When to Use	Next Step
**Mini-Cog**	3–5 min	Very brief; easy to administer	Inhaler errors, inconsistent history, and nonadherence	Perform MoCA or refer; adapt care
**GPCOG**	4–6 min	Includes informant input	Caregiver concerns or memory complaints	Further evaluation or referral
**MoCA**	7–10 min	Sensitive to mild impairment	After a positive brief screen or subtle deficits	Integrate findings; consider referral
**MMSE**	7–10 min	Widely familiar	Where institutional familiarity exists	Confirm borderline results if needed

This table summarises brief cognitive screening tools suitable for time-constrained respiratory consultations. Tools are presented by administration time, key strengths, suggested clinical triggers in COPD, and recommended next steps. These instruments support initial recognition and decision-making and do not replace a comprehensive neuropsychological assessment when indicated. Abbreviations: MMSE—Mini-Mental State Examination, MoCA—Montreal Cognitive Assessment, GPCOG—General Practitioner Assessment of Cognition, COPD—Chronic Obstructive Pulmonary Disease.

**Table 2:** Conceptual distinctions between knowledge, competence, and clinical confidence in cognitive management for COPD.

Concept	Definition (as Used in This Review)	Role in Cognitive Management
Knowledge	Awareness and understanding of cognitive impairment, its prevalence, mechanisms, and clinical implications	Enables recognition of relevance but does not ensure action
Competence	Ability to integrate knowledge, skills, and judgement to perform cognitive assessment and management	Provides the capability to act when required
Clinical confidence	Clinician’s perceived ability to recognise, assess, communicate, and manage cognitive impairment under uncertainty	Determines whether competence is translated into clinical action
Clinical practice	Observable assessment, communication, and management behaviours in routine care	Outcome influenced by confidence-mediated decision-making

This table outlines the roles of knowledge, competence, and clinical confidence, highlighting clinical confidence as the key intermediary between capability and clinical action in COPD management.

**Table 3:** Applied Example of Confidence-Mediated Decision-Making in COPD Cognitive Care.

Clinical Element	Observation	Confidence-Related Issue	Resulting Practice Pattern	Potential Support Mechanism
**Repeated inhaler misuse**	Persistent incorrect technique despite prior education	Hesitation to initiate cognitive screening	Education repeated without adaptation	Use of a brief screening tool (e.g., MoCA), workflow prompt
**Suspected executive dysfunction**	Inconsistent adherence and history	Uncertainty about interpretation and referral pathways	No caregiver involvement; no referral initiated	Defined referral pathway; interdisciplinary collaboration
**Time-constrained consultation**	Competing respiratory priorities	Low confidence in managing cognitive findings within the visit	Cognitive concerns deferred	Structured cognitive trigger checklist

This table presents an illustrative clinical scenario demonstrating how uncertainty and limited clinical confidence may influence cognitive assessment decisions in COPD care. It links observed practice patterns to potential confidence-related barriers and corresponding structural supports.

**Table 4:** Factors Influencing Clinical Confidence and Cognitive Assessment Practices in COPD.

Factor	Confidence Impact	Clinical Effect	Potential Facilitator
**Limited training in cognitive assessment**	Uncertainty in interpretation and communication	Avoidance or selective screening	Structured respiratory-specific cognitive training
**Time constraints during consultations**	Perceived inability to address cognition adequately	Deferred or omitted screening	Brief screening tools; workflow prompts
**Diagnostic ambiguity (mild cognitive impairment)**	Fear of misclassification	Reliance on informal judgement	Clear screening thresholds and referral criteria
**Guideline ambiguity**	Perception that cognitive assessment is optional	Inconsistent integration into routine care	Explicit guideline recommendations
**Role uncertainty across disciplines**	Unclear ownership of screening and follow-up	Deferral to other services	Defined interdisciplinary roles and shared-care pathways
**Organisational limitations**	Limited access to follow-up services	Fragmented referral and poor continuity	Integrated referral systems and documentation standards

This table summarises key barriers that may undermine clinician confidence in cognitive assessment within COPD care, their impact on practice patterns, and corresponding facilitators that may support consistent implementation.

## Data Availability

No new data were created or analysed in this study.
